# Evaluation of HbA1c screening during outreach events for prediabetes subject recruitment for clinical research

**DOI:** 10.1186/s13063-018-2459-0

**Published:** 2018-01-22

**Authors:** Sabina Paglialunga, Ryan Bond, Sharon H. Jaycox

**Affiliations:** Celerion, Global Clinical Research, 2420 W Baseline Rd, Tempe, AZ 85283 USA

**Keywords:** Hemoglobin A1c, Early clinical studies, Clinical research organization, Minority populations, Hispanic recruitment

## Abstract

**Background:**

There are a number of obstacles which may impede the recruitment of underserved populations in clinical research studies; some of these factors include mistrust of medical research, socioeconomic constraints, cultural factors, and language barriers. For chronic metabolic disease indications, these barriers may also include lack of disease awareness. Recently, national organizations such as the American Diabetes Association (ADA) and Centers for Disease Control and Prevention (CDC) have highlighted the need for prediabetes recognition. Therefore the aim of the study was twofold: to raise prediabetes awareness in an under-represented Hispanic community and to engage prediabetes participants in clinical research.

**Methods:**

Hemoglobin A1c (HbA1c) screening was performed at major outreach events catered to the Hispanic community. All participants signed an ethics review board approved waiver which collected basic demographic information and the HbA1c test was performed with a hand-held monitor and finger-stick blood sample. Participants were given their HbA1c results at the event as well as information on prediabetes and upcoming clinic studies. After the event, participants were contacted by a study participant recruiter to assess interest in participating in clinical research.

**Results:**

The majority of participants screened fell within a prediabetes HbA1c range. Mean HbA1c was similar among men and women, yet higher in individuals aged 45–65 years compared to adults aged < 45 years (*p* < 0.05). For recruitment purposes, the highest number of leads came from participants attending a faith-based community event. In all, 17% of individuals contacted expressed interest in participating in clinical research and created a profile within our database to be eligible for future studies.

**Conclusions:**

Providing no-cost HbA1c screening is an excellent recruitment tool for clinical research as well as an opportunity to raise prediabetes awareness in a traditionally underserved population.

## Background

The Food and Drug Administration (FDA) encourages pharmaceutical and biotech companies to enroll participants in clinical trials that are representative of individuals who will likely benefit from the therapeutic drug once approved by the agency. Although a more common practice in late stage clinical trials, a recent trend in this domain is to engage patients early in drug development Phase I and Phase II studies to obtain signals of efficacy in a population of interest. For chronic metabolic disease indications such as type 2 diabetes, this patient population may also include individuals with diabetes risk factors such as obesity and prediabetes.

The incidence of type 2 diabetes and prevalence of these risk factors are commonly observed in under-represented groups such as Hispanics, African Americans, and Native Americans [1]. A number of studies have shown that genetic, environmental, and socioeconomic factors can all contribute to metabolic diseases in disparate populations [2, 3]. While in the US racial and ethnic minorities comprise roughly 40% of the population [4], the number of disparate individuals enrolling in clinical trials is incredibly low [5]. Despite initiatives to increase minority participation in publicly funded medical research such as the Revitalization Act, which was passed in the early 1990s [6], the National Institutes of Health (NIH) continues to see low minority enrollment rates in clinical studies [7]. For instance, African Americans represent 13.2% of the US population yet it is estimated that only 5% participate in clinical studies. Moreover, Hispanics represent 16% of the general population and only 1% participate in clinical trials [8]. Further, in 2012 it was reported that minority participation rates in industry-led clinical studies was estimated to be relatively low at 16.7% [9].

Common barriers to the recruitment of an underserved population in medical research include: a mistrust of medical research; lack of awareness of available studies; economic constraints (i.e. loss of wages, need for childcare); language barriers; lack of transportation; and failure to meet inclusion criteria [10, 11]. To address these challenges, attention should be focused on education of medical research programs with support from community entities. For metabolic disease research lines, active community-based recruitment has proven to be a successful strategy [12]. Within the Hispanic community, there are organized health fairs, cultural events, and religious centers that can serve as integral recruitment sources. The aim of this study was to examine the recruitment efficiency of prediabetes and type 2 diabetes participants at various community events marketed to a Hispanic population. Furthermore, to increase prediabetes and type 2 diabetes awareness in a traditionally underserved community, we provided free hemoglobin A1c (HbA1c) screening. HbA1c reflects average blood glucose over the past three months and can be used to identify individuals with prediabetes and type 2 diabetes.

## Methods

We attended five major events catered to a Hispanic population from May to September 2016 in Phoenix, Arizona: two health fairs, a church open-house, a cultural street party, and a county exposition. The event organizers were responsible for advertising the events to the community which included but was not limited to flyers, billboards, TV, radio, social media, and print advertisements. Celerion participated as an exhibitor at these events.

All participants signed a waiver form approved by an ethics review board. Basic demographic data were collected, such as date of birth and ethnicity. Participants were given an explanation of the procedure as well as literature on HbA1c and prediabetes. English and Spanish material as well as English- and Spanish-speaking staff were available at all events. The personnel that conducted the screening included nurses, study participant recruiters (SPR), and metabolic specialists. The screening was performed using a hand-held HbA1c monitor (PTS Diagnostics, Indianapolis, IN, USA) with a finger-stick blood sample. The HbA1c monitor has a coefficient of variation of 4.59% and 5.31% for a low and high glycated-hemoglobin control solution (Nova-One Diagnostic, Calabasas, CA, USA), respectively. HbA1c results were obtained within 5 min and reported to the participant. Following the ADA guidelines [13], HbA1c values were categorized as healthy (<5.7%; 39 mmol/mol), prediabetes (5.7–6.4%; 39–46 mmol/mol), and diabetes (≥ 6.5%; 48 mmol/mol). When HbA1c values were outside of the healthy range, the participant was encouraged to contact their primary care physician for a diagnosis; primary care provider information was available for those without a family practitioner.

After the event, a Celerion SPR made two attempts to contact all screened participants to assess interest in participating in clinical research and documented responses. The goal of the follow-up calls was to register interested individuals into our participant database for future paid medical research studies.

### Statistical analysis

Age and HbA1c results are presented as mean and standard deviations (SD), with ranges and sample size (*n*) given. Statistically significant differences were analyzed by unpaired Student’s t-test or one-way ANOVA followed by Tukey’s post-hoc test as indicated in the figure or table legend. Categorical data such as gender, ethnicity, and diabetes status are presented as sample size (*n*) and percentage (%) with statistical significance determined by Chi-squared test with degrees of freedom (df) shown (χ^2^_df_). Statistical significance set at *p* < 0.05.

## Results

A total of 401 participants expressed an interest in the event and 391 participated in the free HbA1c screening. Across all five events, more women signed up for the screening than men, approximately 60% vs 40% (Table 1). The individuals at the church event were, on average, older than those participating at the health fairs or other community events. By design, the majority of participants were of Hispanic descent. Hispanic ethnicity was almost exclusively indicated by participants attending the two health fairs, with < 5% indicating “other” or “unknown.” Meanwhile, a more diverse population attended the other community events (Table 1).

**Table 1:** Participant demographic information and type 2 diabetes prevalence

Characteristic	Total	Health fair 1	Health fair 2	Church event	Street party	County expo	Difference
Age (years)							
Mean (SD)	46.3 (13.5)	46.0 (13.0)	44.7 (13.0)**	51.2 (13.9)	43.3 (15.0)**	45.8 (11.9)	*p* = 0.0029
Range	18–100	18–90	18–76	28–100	18–94	21–73	
*n*	401	61	99	86	62	93	
Gender, *n* (%)							
Male	157 (39.4)	24 (39.3)	40 (40.0)	34 (40.5)	24 (38.7)	35 (38.5)	χ^2^_4_ = 0.1014,
Female	242 (60.6)	37 (60.7)	60 (60.0)	50 (59.5)	38 (61.3)	56 (61.5)	*p* = 0.9988
Ethnicity, *n* (%)							
Hispanic	308 (78.8)	58 (95.1)	95 (100.0)	47 (54.7)	40 (64.5)	68 (78.2)	χ^2^_4_ = 72.78,
Non-Hispanic	84 (21.5)	3 (4.9)	0 (0.0)	39 (45.3)	22 (35.5)	19 (21.8)	*p* < 0.0001
HbA1c (%)							
Mean (SD)	6.0 (1.4)	6.4 (1.7)*	6.0 (1.5)	5.7 (0.7)	6.2 (1.3)	5.9 (1.4)	*p* = 0.0259
Range	4.5–13.9	5.1–13.0	4.6–12.3	4.9–10.2	4.9–11.9	4.5–13.9	
*n*	391	60	94	86	61	90	
Prevalence, *n* (%)							
Healthy	204 (52.2)	25 (41.7)	56 (59.6)	46 (53.5)	26 (41.9)	53 (58.2)	χ^2^_8_ = 17.61,
Prediabetes	129 (33.0)	21 (35.0)	22 (23.4)	33 (38.4)	23 (37.1)	30 (33.0)	*p* = 0.0243
Type 2 diabetes	58 (14.8)	14 (23.3)	16 (17.0)	7 (8.1)	13 (21.0)	8 (8.8)	

Age and HbA1c were analyzed by one-way ANOVA followed by Tukey’s post-hoc test where *p < 0.05, **p < 0.01 vs Church event

Gender, ethnicity, and prevalence rates were analyzed by Chi-squared test

**Fig. 1:**
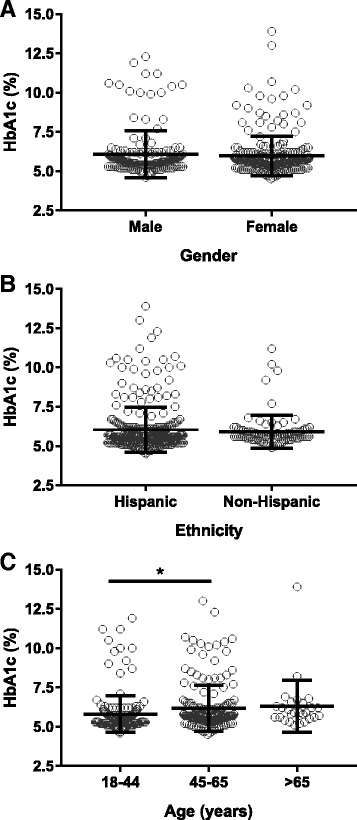
HbA1c values obtained during outreach events. HbA1c relative to (**a**) gender, (**b**) ethnicity, and (**c**) age. Data presented as *scatter plot* of individual values (*circles*) with mean (*middle black line*) and standard deviation (*upper and lower black lines*). Gender and ethnicity were analyzed by an unpaired Student’s t-test and age was analyzed by a one-way ANOVA followed by a Tukey’s post-hoc test, where * indicates p < 0.05 vs 18–44-year age group

The average HbA1c value for all participants screened was 6.0 ± 1.4%, which falls within the prediabetes range, indicating a high risk of insulin resistance among this study population. HbA1c was similar among male and female individuals (Fig. 1a) as well as Hispanic and non-Hispanic participants (Fig. 1b); however, it was significantly higher in the 45–65-year age group (*p* < 0.05, Fig. 1c). The overall prevalence of prediabetes and type 2 diabetes, based on HbA1c values, were 33.0% and 14.8%, respectively. The highest incidence of prediabetes was observed at the church and cultural street party events, with ~ 38% each. Furthermore, type 2 diabetes was more common at health fair 1, observed in 23% of the participants (Table 1).

**Fig. 2:**
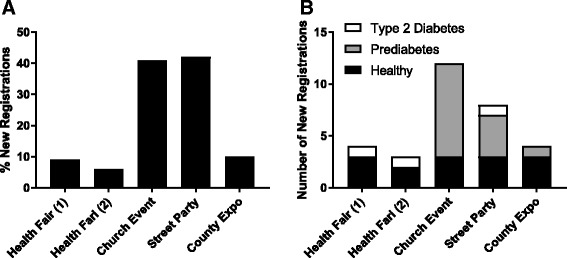
Number of participants registered to participate in clinical studies through outreach events. (**a**) Percentage of individual registration during SPR calls by event. (**b**) Number of healthy, prediabetes, and type 2 diabetes registered participants by event

Following these events, a SPR was able to reach 187 individuals by phone to inquire about interest in participating in clinical research. The contact rate was in the range of 31–78%, with leads generated from the two health fairs having the greatest success in reaching the participants by phone. Overall, from the responses collected at the event and during the outreach calls, 14% of individuals stated they were interested in participating in clinical research, while 47% were not (Table 2). In addition, 19% expressed they would call back at a later time, 7% indicated they would go online to create a registration profile, and 2% requested more information about clinical research participation and available studies. Reasons for not registering during the outreach call included; scheduling issues; not having proper identification; or wanting to discuss with family members first. A total of 31 participants (17% of all individuals contacted by phone) created a profile within our database to be able to participate in future clinical studies. It is interesting to note that four individuals created profiles after the initial contact from our SPR team, having time to review our website and information provided about clinical study participation. The church event was the most successful for recruiting followed by the street party and the county expo (Fig. 2a). Leads from the two health fairs resulted in the lowest recruitment rate. In all, 14 prediabetes and three type 2 diabetes individuals were added to our database through this recruitment initiative (Fig. 2b).

**Table 2:** Gauging interest in clinical research participation

Response	Total	Health fair 1	Health fair 2	Church event	Street party	County expo
Contacted, *n* (%)	187 (47.8)	47 (78.3)	51 (54.3)	29 (33.7)	19 (31.1)	41 (45.6)
Responses:						
Interested, *n* (%)	26 (13.9)	6 (12.5)	1 (2.0)	9 (25.0)	6 (24.0)	4 (9.5)
Not interested, *n* (%)	88 (47.1)	13 (27.1)	29 (56.9)	10 (27.8)	12 (48.0)	24 (57.1)
Will call back, *n* (%)	36 (19.3)	14 (29.2)	12 (23.5)	3 (8.3)	2 (8.0)	5 (11.9)
Will go online, *n* (%)	14 (7.5)	8 (16.7)	2 (3.9)	2 (5.6)	2 (8.0)	0
Scheduling issues, *n* (%)	11 (5.9)	2 (4.2)	1 (2.0)	8 (22.2)	0	0
No ID, *n* (%)	9 (4.8)	5 (10.4)	3 (5.9)	0	0	1 (2.4)
To discuss with family, *n* (%)	2 (1.1)	0	0	0	0	2 (4.8)
Requested info, *n* (%)	4 (2.1)	0	2 (3.9)	0	0	2 (4.8)
Other, *n* (%)	12 (6.4)	0	1 (2.0)	4 (11.1)	3 (12.0)	4 (9.5)

## Discussion

The HbA1c screening during outreach events serves two key objectives: raising type 2 diabetes and prediabetes awareness in under-represented communities and assessing interest in paid medical research participation. In the US, an estimated 9.4% of the adult population has type 2 diabetes [14]. This national average is also similar for the state of Arizona, with an estimated 9.1% of the population diagnosed with diabetes [15]. However, here we observed 14.8% of participants falling within the diabetes range based on the HbA1c screening. We did not collect medical history information at the time of the outreach event and are not able to report the percent of individuals with a known diabetes diagnosis. Both the ADA and CDC recommend that screening for undiagnosed diabetes should be managed by a healthcare organization, as they express concerns that community-based screening programs tend to have low yield and poor follow-up, preferring efforts and resources directed to intervention programs [16]. Nonetheless, it is important to note that one-third of Americans have prediabetes, yet only 10% are aware of their condition [14]. Taken altogether, these alarming statistics stress the importance of screening events regardless of the sponsoring organization.

Coupling lack of prediabetes awareness with little education regarding clinical studies leads to a very narrow overlap of potential participants; the result is a common challenge for clinical study success and participant recruitment [17]. To remedy this, several avenues of solicitation exist such as TV, radio, printed advertisements, and social media; and clinical research organizations do not rely solely on one medium. While handing out flyers can be an ineffective recruitment strategy [12, 18–20], numerous studies have shown that community-based programs can be highly effective to engage disparate populations in clinical research [21, 22]. From a recruitment perspective, we determined that cultural and church events were more successful in registering individuals for paid clinical studies than events marketed as “health fairs.” Indeed, the effectiveness of using churches for community outreach recruiting is well documented [12, 23]. One potential reason for this difference in registration rate observed here might be related to the participants’ expectations. For the most part, health fair attendees were strictly seeking medical services. The two health fairs were designed to support the wellbeing and health of an underserved population, providing a variety of free medical services such as dental health checks, blood pressure testing, and HIV and pregnancy screening. On the other hand, the community events offered traditional food, music, entertainment, and shopping in a social setting, which may have provided an atmosphere for individuals willing to participate in research. This small group approach has also been noted to be more effective in other instances of recruitment for difficult to reach populations in contrast to the traditional one-on-one delivery [24]. Research by Ramesy et al. found referrals from friends provided a boon to recruitment with difficult-to-recruit groups [25]. Given the familial nature of interpersonal community events, it should not be unexpected that the relationship between people might drive interest or referral for others once communal education is presented. This was noted within our study, multiple individuals of the same family participated in the screen (personal observation). This group effort provides a unique opportunity for recruitment along with education. While someone with the condition may not be present, given the prevalence of diabetes, education of a potential family member at the very least can drive a later conversation which could result in treatment or enrollment depending on circumstances.

There are a number of reasons why people are interested in participating in clinical research. Geppert et al. recently described that altruism and receiving (better) treatment are chief motivations for diabetes participants who are willing to participate in clinical research. Concerns over risk and aversion to research were cited for those not willing to participate [26]. Robiner et al. also identified economic burden (missing work), study schedule (study length and frequency of clinic visits), as well as procedural discomfort as major barriers to participating in a diabetes clinical study [27]. Although here we were not recruiting for a particular study, we were able to gauge the main reason for not registering within our database was unwilling/not interested in clinical research. Other deterrents to registration included scheduling issues and lack of proper identification. Also, many participants were not aware that clinical studies are available for those who do not fall within the “normal healthy” category, such as obese, prediabetes, and type 2 diabetes individuals. Providing education regarding clinical studies and inclusion/exclusion criteria during the screening may encourage more people to participate in clinical research in the future.

A number of study limitations must be addressed. Lack of medical history, anthropometric, or clinical data other than HbA1c limits the characterization of the study population to simple demographic analysis. While, we attempted to capture information why individuals were not willing to participate in clinical research, we did not record the reasons others did register. Understanding both the needs and concerns of potential participants may provide valuable insight for future recruitment strategies. In addition, only two follow-up calls were made to individuals to engage in recruitment discussions; these were made shortly after the event was held. Further contact with potential participants may have increased recruitment rates.

## Conclusion

The HbA1c screening is an effective recruitment tool, with one new registration for every 13 participants screened. Furthermore, offering free HbA1c screening at local health fairs and cultural events can provide an underserved population with a valuable medical service and is an excellent opportunity to raise diabetes awareness and provide education on clinical studies.

## Abbreviations

ADA: American Diabetes Association
CDC: Centers for Disease Control and Prevention
FDA: Food and Drug Administration
HbA1c: Hemoglobin A1c
HIV: Human immunodeficiency virus
SPR: Study participant recruiter
US: United States of America
